# Identification of Host Trafficking Genes Required for HIV-1 Virological Synapse Formation in Dendritic Cells

**DOI:** 10.1128/JVI.01597-19

**Published:** 2020-04-16

**Authors:** Rebecca Bayliss, James Wheeldon, Stephan M. Caucheteux, Carien M. Niessen, Vincent Piguet

**Affiliations:** a Division of Infection and Immunity, School of Medicine, Cardiff University, Cardiff, United Kingdom; b Department of Medicine, University of Toronto, Toronto, Canada; c Department of Dermatology, Cologne Excellence Cluster on Cellular Stress Responses in Aging Associated Diseases (CECAD), and Center for Molecular Medicine, University of Cologne, Cologne, Germany; d Division of Dermatology, Women’s College Hospital, Toronto, Canada; e Division of Dermatology, Department of Medicine, University of Toronto, Toronto, Canada; Ulm University Medical Center

**Keywords:** dendritic cell, HIV-1, T-cell immunity, virological synapse, host-cell interactions

## Abstract

Dendritic cells (DCs) are one of the earliest targets of HIV-1 infection acting as a “Trojan horse,” concealing the virus from the innate immune system and delivering it to T cells via virological synapses (VS). To explicate how the virus is trafficked through the cell to the VS and evades degradation, a high-throughput small interfering RNA screen targeting membrane trafficking proteins was performed in monocyte-derived DCs. We identified several proteins including BIN-1 and RAB7L1 that share common roles in transport from endosomal compartments. Depletion of target proteins resulted in an accumulation of virus in intracellular compartments and significantly reduced viral *trans*-infection via the VS. By targeting endocytic trafficking and retromer recycling to the plasma membrane, we were able to reduce the virus’s ability to accumulate at budding microdomains and the VS. Thus, we identify key genes involved in a pathway within DCs that is exploited by HIV-1 to traffic to the VS.

**IMPORTANCE** The lentivirus human immunodeficiency virus (HIV) targets and destroys CD4^+^ T cells, leaving the host vulnerable to life-threatening opportunistic infections associated with AIDS. Dendritic cells (DCs) form a virological synapse (VS) with CD4^+^ T cells, enabling the efficient transfer of virus between the two cells. We have identified cellular factors that are critical in the induction of the VS. We show that ADP-ribosylation factor 1 (ARF1), bridging integrator 1 (BIN1), and Rab GTPases RAB7L1 and RAB8A are important regulators of HIV-1 trafficking to the VS and therefore the infection of CD4^+^ T cells. We found these cellular factors were essential for endosomal protein trafficking and formation of the VS and that depletion of target proteins prevented virus trafficking to the plasma membrane by retaining virus in intracellular vesicles. Identification of key regulators in HIV-1 *trans*-infection between DC and CD4^+^ T cells has the potential for the development of targeted therapy to reduce *trans*-infection of HIV-1 *in vivo*.

## Abstract (precis)

The lentivirus human immunodeficiency virus (HIV) targets and destroys CD4^+^ T cells, leaving the host vulnerable to life-threatening opportunistic infections associated with AIDS. Dendritic cells (DCs) form a virological synapse (VS) with CD4^+^ T cells, enabling the efficient transfer of virus between the two cells. We have identified cellular factors that are critical in the induction of the VS. We show that ADP-ribosylation factor 1 (ARF1), bridging integrator 1 (BIN1), and Rab GTPases RAB7L1 and RAB8A are important regulators of HIV-1 trafficking to the VS and therefore the infection of CD4^+^ T cells. We found these cellular factors were essential for endosomal protein trafficking and formation of the VS and that depletion of target proteins prevented virus trafficking to the plasma membrane by retaining virus in intracellular vesicles. Identification of key regulators in HIV-1 *trans*-infection between DC and CD4^+^ T cells has the potential for the development of targeted therapy to reduce *trans*-infection of HIV-1 *in vivo*.

## INTRODUCTION

Dendritic cells (DCs) are key antigen-presenting cells that provide an important link between innate and adaptive immune systems, activating T cells (reviewed in references 1 and 2). Although HIV-1 is able to replicate in DCs, the process is inefficient and produces low levels of infectious virus (3–8). However, DCs are able to transfer intact viral particles to target T cells via virological synapses (VS) by a process termed “*trans*-infection” (9), contributing to the spread of infection *in vivo* (10, 11).

HIV-1 *trans*-infection has been shown to depend on the ability of the virus to “surf” along the surface of the DC via actin-rich dendrites to promote *trans*-infection (12–14). Several studies conducted in macrophages and DCs located viruses sequestered into plasma membrane invaginated compartments from which viral particles are released at the VS (15–18). These compartments are thought to be surface accessible (15); however, there is evidence of a population becoming isolated from the cell surface (16). It is established in macrophages and DCs that these surface-accessible compartments may have complex morphologies that require membrane trafficking regulation, such as the virus-containing compartments found in macrophages (17).

In contrast, *cis*-infection of DCs is limited by the host restriction factor SAMHD1, a dinucleotide triphosphate hydrolase that blocks reverse transcription of viral DNA (19–24). In addition, viral cytosolic DNA is sensed by cGAS, a GMP-AMP synthase that induces an interferon type I response in DCs (25–27) restricting productive viral replication.

It has been previously reported that HIV-1 enters the cell through the endolysosomal pathway, with evidence supporting roles for clathrin-mediated endocytosis (4, 28, 29), receptor-mediated endocytosis (30, 31), and macropinocytosis (32). However, at later time points virus accumulates in virus-containing compartments rich in tetraspanins, such as CD81, that are continuous with the plasma membrane (4, 15, 17). More recent studies identified the importance of tetraspanin 7 (TSPAN7) and dynamin 2 (DNM2) in maintaining viral particles on dendrites and promoting efficient viral transfer. Disruption of these targets led to sequestration of virus in intracellular vesicles and a reduction in viral transfer (13).

To elucidate the role of membrane trafficking in the capture and trafficking of the virus through DCs to the VS, we performed a high-throughput small interfering RNA (siRNA) screen targeting membrane trafficking proteins. Our results identified proteins involved in vesicle trafficking between early endosomes, the *trans*-Golgi network (TGN), and the plasma membrane that reduce transfer of HIV-1 from DC to T cells. We show that HIV-1 is dependent on a functioning endocytic pathway, the disruption of which results in an accumulation of virus in intracellular vesicles, blocking trafficking of the virus to the virological synapse.

## RESULTS

### The siRNA membrane trafficking library identified genes involved in HIV-1 *trans*-infection between DCs and T cells.

**FIG 1:**
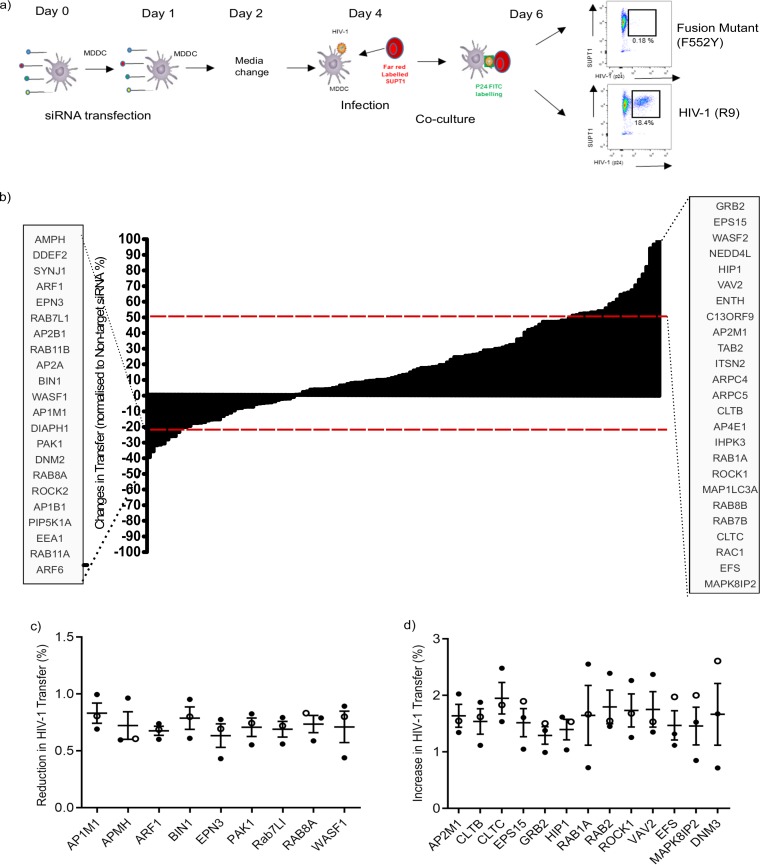
Procedure and results of the siRNA screen used to investigate *trans*-infection of HIV-1 between MDDCs and T cells. (a) Schematic of the method used to study the effects of siRNA knockdown on HIV-1 *trans*-infection between DCs and CD4^+^ T cells. (b) Results of siRNA screen on HIV-1 *trans*-infection between MDDCs and CD4^+^ T cells. Red dashed lines indicate the assay cutoffs of –20% and +50% for nonspecific variation of the assay. siRNA that reduced or increased HIV-1 *trans*-infection above or below the cutoff point (HITS) are listed in the gray boxes. (c) Identification of genes that reduced HIV-1 *trans*-infection between MDDCs and T cells. Results from initial screen conducted in SUPT1 cells (○) are shown in combination with repeats conducted in with autologous CD4^+^ T cells (●). Means and standard deviations (SD) of three independent donors shown. Only genes with a mean percentage below that of the nontarget siRNA are shown. (d) Identification of genes that increase HIV-1 *trans*-infection between MDDCs and T cells. Results from initial screen conducted in SUPT1 (○) cells are shown in combination with two repeats conducted with autologous CD4^+^ T cells (●). The means and SD for three independent donors were calculated per gene. Only genes with a mean above that of the nontarget siRNA are shown.

To identify the cellular trafficking pathways involved in the transfer of HIV-1 in *trans*-infection from DC to CD4^+^ T cells, an siRNA library targeting 140 membrane trafficking genes was utilized. SMARTpool siRNA was transfected into monocyte-derived DCs (MDDCs) 48 h before infection with full-length CXCR4-tropic HIV-1 (R9) and cocultured with SUPT1 cells at 1:1 ratio. HIV-1-infected SUP-T1 cells were analyzed by flow cytometry 48 h later. No infection was detected in SUP-T1 cells inoculated with an HIV-1 fusion-mutant control (Fig. 1a). Nontarget siRNA was used to compare infection levels and showed <20% variation between replicates (Z score = 1.5 standard deviation); therefore, the lower assay cutoff point was set at 20%.

In the primary screen, the knockdown of 16 genes induced a reduction in HIV-1 *trans*-infection of ≥20%, whereas 25 genes showed an increase in viral *trans*-infection by >50% (Fig. 1b). The primary Hits were reproduced in two donors using autologous CD4^+^ T cells activated with interleukin-2 (IL-2) and phytohemagglutinin (PHA). Nine hits showed a reproducible reduction in HIV-1 transfer: *AP1M1*, *AMPH1*, *ARF1*, *BIN1*, *EPN3*, *PAK1*, *RAB7L1*, *RAB8A*, and *WASF1* (Fig. 1c). Hits that resulted in an increase in HIV-1 transfer included *AP2M1*, *CLTB*, *CLTC*, *EPS15*, *GRB2*, *HIP1*, *RAB1A*, *RAB2*, *ROCK1*, *VAV2*, *EFS*, *MAPK8IP2*, and *DNM3* (Fig. 1d).

### Efficient HIV-1 *trans*-infection requires vesicle trafficking at the plasma membrane.

To understand potential relationships between the genes selected in the siRNA screen, gene annotation enrichment analysis was used to identify common interactions between the candidate genes that may be involved in the *trans*-infection of HIV-1 between DCs and T cells. Analysis of the siRNA candidates was carried out for cellular compartments and biological processes (Table 1). Our results show that the genes required for optimal viral transfer are primarily involved in endocytic compartment regulation, whereas genes that restrict viral transfer are largely involved in clathrin-coat-mediated endocytosis and actin-dependent processes at the plasma membrane. Taken together, the data suggest that preventing viral uptake via clathrin-coated vesicles enhances viral transfer, which is likely due to increased retention of virus on the cell surface. This finding is in agreement with studies that show HIV-1 transmitted in *trans* between DCs and T cells from the surfaces of DCs (12, 13). In contrast, genes required for efficient *trans*-infection are strongly associated with cytoplasmic membrane-bound vesicles and vesicle-mediated transport, supporting the view that HIV-1 is sequestered into intracellular virus-containing compartments (VCC) (15, 16, 33).

**TABLE 1:** Network analysis statistical data

Gene category and nodename	*P*	*q*	Dataset size
Cellular components: facilitating genes^*a*^			
Cytoplasmic membrane-bound vesicle	1.781257032788977E–5	0.0026505776165784978	7
Cytoplasmic vesicle	2.9736862512104874E–5	0.0022129769452692294	7
Golgi apparatus	7.260275955618954E–5	0.0035995739100488366	7
Clathrin-coated vesicles	1.1280567574068089E–4	0.004193431353399224	4
*trans*-Golgi network	1.5015004674908726E–4	0.004464810314555923	4
			
Cellular compartments: inhibitory genes^*b*^			
Clathrin coat of coated pit	1.98808829214536E–8	2.0278480239444008E–6	4
Clathrin vesicle coat	7.728295638241832E–8	3.9414231596257565E–6	4
Clathrin coat	5.75990999192209E–7	1.9583507854470383E–5	4
Vesicle coat	6.170337924836827E–7	1.5734242778675522E–5	4
Coated pit	1.8932548517714428E–6	3.86216897007019E–5	4
			
Biological processes: facilitating genes^*c*^			
Vesicle-mediated transport	2.5476631776993885E–7	1.4444208784170076E–4	8
Membrane orgainization	7.045745098037326E–5	0.019777204754682143	6
Endocytosis	1.3846610717114176E–4	0.025832390294862395	5
Vesicle organization	2.614347913818171E–4	0.03638479278473983	4
Cellular protein localization	2.906708321191288E–4	0.03242937937435153	6
			
Biological processes: inhibitory genes^*d*^			
Vesicle-mediated transport	2.60964598927977E–5	0.01146851519073222	6
Endocytosis	3.158300588098925E–5	0.006955651219872405	5
Receptor-mediated endocytosis	1.0101208431475834E–4	0.0147729914135587	4
Establishment of protein localization	1.375341659809643E–4	0.015083655172842936	6
Protein localization	3.4173910866504216E–4	0.02976299308721586	6

a Statistical data (*P* and *q* values) are presented for cellular compartments of genes facilitating HIV-1 *trans*-infection. Values of <0.005 are displayed for each node name. The number of data sets included in the process is indicated under the data set size heading.

b Statistical data (*P* and *q* values) are presented for cellular compartments of genes inhibitory to HIV-1 *trans*-infection. Values of <0.005 are displayed for each node name. The number of data sets included in the process are indicated under the data set size heading.

c Statistical data (*P* and *q* values) are presented for biological processes of genes facilitating HIV-1 *trans*-infection. Values of <0.005 are displayed for each node name. The numbers of data sets associated with the cellular compartments are indicated under the data set size heading.

d Statistical data (*P* and *q* values) are presented for biological processes of genes inhibitory to HIV-1 *trans*-infection. Values of <0.005 are displayed for each node name. The numbers of data sets included in the process are indicated under the data set size heading.

### *ARF1*, *BIN1*, *RAB7L1*, and *RAB8A* are required for HIV-1 *trans*-infection.

The siRNA library used to identify target genes is comprised of a set of four separate siRNA sequences that target different regions of the same gene; these are pooled to reduce the potential off-target effects of siRNA transfection. The knockdown of the pooled siRNA typically reflects the most functional siRNA within the pool. Therefore, the four individual siRNA can be analyzed for their ability to reduce viral transfer to validate whether the observed phenotype is a genuine on-target effect.

**FIG 2:**
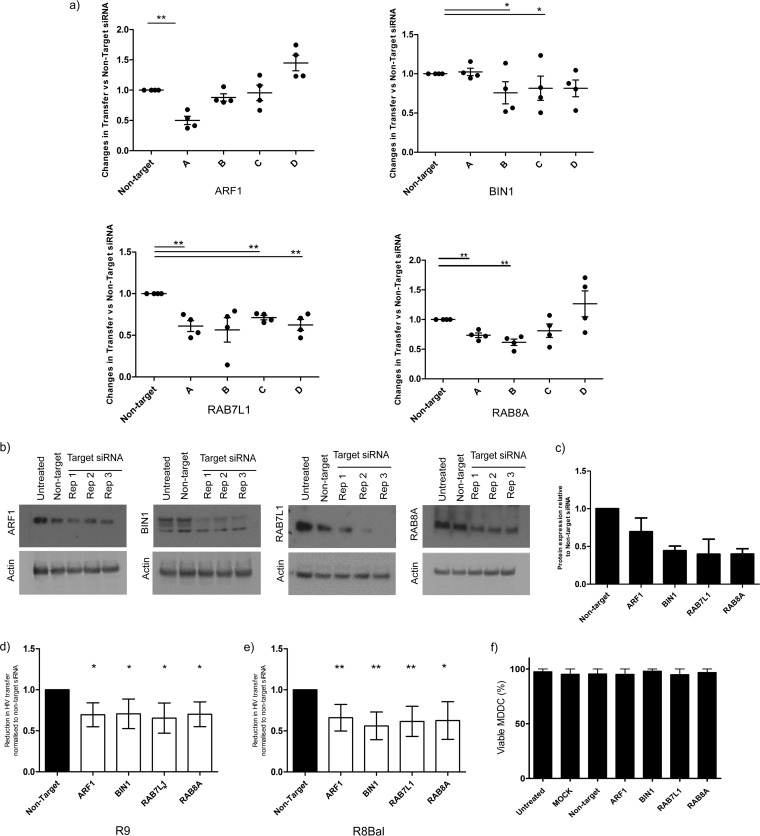
ARF1, BIN1, RAB7L1, and RAB8A regulate HIV *trans*-infection in DCs and T cells. (a) Validation of siRNA knockdown on *trans*-infection against four individual siRNAs from each candidate gene. The percentage of HIV-1 transfer is normalized to nontarget siRNA set at a value of 1.0. Each point represents an individual donor. The means ± the SD are shown. *, *P* < 0.05; **, *P* < 0.005. (b) Western blot analysis of pooled siRNA knockdown in MDDCs at 72 h posttransfection with *ARF1*, *BIN1*, *RAB7L1*, and *RAB8A* siRNA performed in triplicate in untreated MDDCs and non-target siRNA. Actin is used as a loading control. (c) Densitometry quantification of protein expression levels for ARF1, BIN1, RAB7L1, and RAB8A. The protein expression levels for siRNA-transfected MDDCs were normalized to an actin loading control. All values are relative to nontarget siRNA-transfected lanes (set at 1.0). The means ± the SD are shown (*n* = 3). (d) Effects of final target siRNA on HIV-1 *trans*-infection with CXCR4 (R9). The reduction in viral transfer was measured relative to nontarget siRNA. The means and SD are shown for each sample (*n* = 5). *, *P* < 0.05; **, *P* < 0.005. (e) Effects of final target siRNA on HIV-1 *trans*-infection with CCR5 (R8Bal). The reduction in viral transfer was measured relative to nontarget siRNA. The means and SD are shown for each sample (*n* = 5). *, *P* < 0.05; **, *P* < 0.005. (f) The effects of ARF1, BIN1, RAB7L1, and RAB8A siRNA transfection on the viability of MDDCs at 48 h posttransfection. All samples compared to untreated MDDCs. Cell viability is shown as a percentage. The means ± the SD are shown (*n* = 2).

The main aim of our study was to identify cellular pathways involved in the delivery of HIV-1 to the VS to aid *trans*-infection; therefore, we focused our investigation on the genes that facilitate the transfer of HIV-1 between DC and T cells. Each of the four siRNA were transfected individually into MDDCs, infected with HIV-1 (R9) and cocultured with autologous T cells for 48 h. Of the final nine candidates, three showed a reduction in transfer (≥20%) in at least two of the four individual siRNA, across four independent donors: *BIN1* (siRNAs B and C), *RAB7L1* (siRNAs A, C, and D) and *RAB8A* (siRNAs A and B) (Fig. 2a). An average reduction of 50% in transfer was evident for ARF1 siRNA A, whereas ARF1 siRNA B produced a 17 to 20% knockdown in viral transmission in three of the four donors analyzed. Thus, in conjunction with the targeted reduction of ARF1 at the protein level, this result indicates that ARF1 siRNA A was the most functional siRNA in the pool, and it was therefore decided to pursue this candidate further.

To determine the level of protein depletion in MDDCs, cell lysates transfected with 200 nM pooled siRNA (Fig. 2b) targeting the entire length of the gene were analyzed by Western blotting. Knockdown was quantified by densitometry relative to protein expression levels in nontarget siRNA-transfected lysates. An efficient knockdown was achieved using pooled siRNA; a 35% ± 17% reduction in protein expression was observed for ARF1, 52% ± 6.6% for BIN1, 53% ± 23.4% for RAB7L1, and 54% ± 8.1% for RAB8A compared to nontarget siRNA (Fig. 2c).

To confirm whether siRNA is capable of reducing viral *trans*-infection independent of viral strain, MDDCs were transfected with the selected target siRNA and infected with either R8BAL (CCR5-tropic) and R9 (CXCR4-tropic) HIV-1. A significant reduction in viral transfer, ranging between 26 and 40% in R9-infected cells and between 35 and 45% in R8BAL-infected cells, was observed for all candidates, demonstrating that host factors involved in trafficking to the VS are shared for both CXCR4- and CCR5-tropic strains of HIV-1 (Fig. 2d and e). All MDDC transfected with pooled siRNA remained >80% viable compared to control cells, ensuring that the reduction in transfer is not due to the cellular toxicity of the siRNA transfection (Fig. 2f). Further, siRNA transfection of MDDCs resulted in a marginal (<5%) increase in the DC maturation marker CD83. Viral binding of p24 Gag also saw a marginal increase compared to untreated and mock-transfected cells; however, HIV-1 internalization was not affected, confirming that the observed reduction in *trans*-infection is not due to decreased binding or internalization of the virus (data not shown).

Additional experiments were conducted on selected candidate siRNAs showing evidence of protein knockdown and a reduction in viral *trans*-infection in at least two of the four individual siRNAs tested. siRNA candidate genes that failed to meet these criteria (WASF1, EPN3, PAK1, and AMPH1) showed no evidence of a reduction in viral *trans*-infection when MDDCs were transfected with individual siRNAs, nor were we able to detect a specific knockdown in protein expression, suggesting that the previously observed reduction in viral *trans*-infection maybe due to off-target effects of those specific siRNAs. Therefore, these genes were eliminated from further analysis along with AP1M1 which showed high variability in the reduction of viral transfer between donors. The final candidates included ARF1, associated with retrograde transport at the Golgi compartment and protein transport to endosomes (34, 35); BIN-1, known to form a complex with dynamin to control vesicle transport and scission (36); RAB7L1, a GTPase required for retromer recycling between the TGN and endosomes (37); and GTPase RAB8A, which is involved in polarized vesicular trafficking to the plasma membrane from the TGN (38).

### Depletion of target proteins reduces virological synapse formation between MDDC and CD4^+^ T cells.

**FIG 3:**
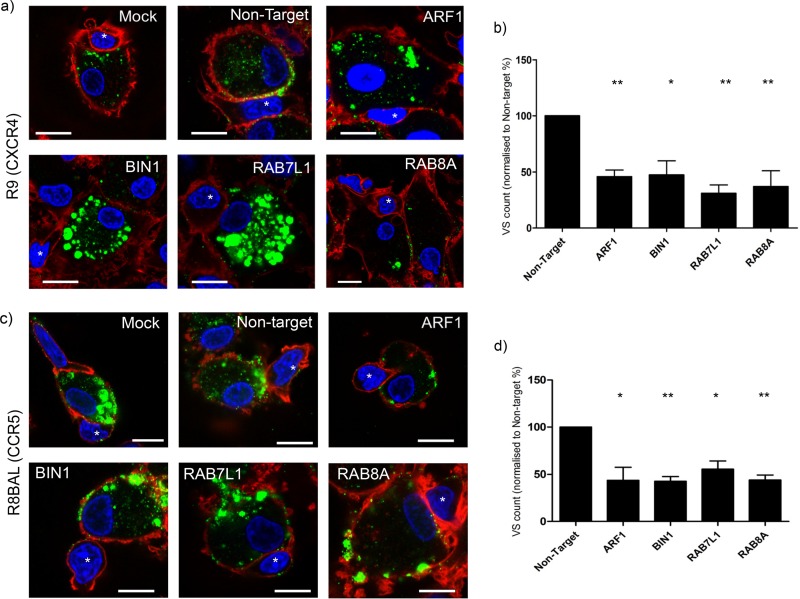
ARF1, BIN1, RAB7L1, and RAB8A are regulators virological synapse formation between HIV-1 infected MDDCs and CD4^+^ T cells. (a) Images of CXCR4 HIV-1 R9 (p24 green)-infected, siRNA-transfected MDDCs interacting with CD4^+^ T cells (identified by an asterisk [*]). Actin, red; nuclei, blue. Scale, 10 μm. (b) Quantification of virological synapse formation between MDDCs and CD4^+^ T cells was counted in siRNA-transfected MDDCs infected with HIV-1 R9 and cocultured with autologous CD4^+^ T cells. T cells are identified as the smaller cells with less cytoplasmic content compared to the larger MDDCs in coculture. Data were normalized to MDDCs transfected with nontarget siRNA. The means and SD for three independent donors (*n* = 500 cells) are shown. *, *P* < 0.05; **, *P* < 0.005. (c) Images of CCR5 HIV-1 R8BAL (p24 green)-infected, siRNA-transfected MDDCs interacting with CD4^+^ T cells (identified by an asterisk [*]). Actin, red; nuclei, blue. Scale, 10 μm. (d) Quantification of virological synapse formation between MDDCs and CD4^+^ T cells was performed in transfected MDDCs infected with HIV-1 R8BAL and cocultured with autologous CD4^+^ T-cells. Data were normalized to MDDCs transfected with nontarget siRNA. The means and SD for three independent donors (*n* = 300 cells) are shown. *, *P* < 0.05; **, *P* < 0.005.

The efficient *trans*-infection of HIV-1 from DCs to T cells is dependent on the formation of VS, an adhesive structure that promotes viral transmission (39, 40). To assess whether the observed reduction in *trans*-infection was due to a reduction in VS formation, siRNA-transfected MDDCs were infected with HIV-1 R9 or R8BAL and cocultured with autologous CD4^+^ T-cells. Imaging of the transfected MDDCs revealed that in the case of *BIN1* and *RAB7L1* siRNA-transfected cells, HIV-1 R9 appeared to accumulate in large cellular vesicles at the plasma membrane and did not form VS with the T cells in spite of apparent interactions between the two cell types. In addition, *ARF1*- and *RAB8A*-depleted cells also appear to inhibit VS formation; however, the accumulation of virus can be seen in smaller vesicles at the cell periphery (Fig. 3a). Quantification of VS was similar in nontarget siRNA, untreated, and mock-transfected cells. All candidate siRNAs had a 40 to 60% reduction in VS formation between DCs and T cells compared to nontarget siRNA-transfected cells (Fig. 3b). Similar results were seen for R8BAL-infected MDDCs, where a reduction in VS number with T cells was observed; however, *BIN1*- and *RAB7L1*-transfected cells did not accumulate virus in intracellular vesicles to the extent seen in R9-infected MDDCs (Fig. 3c and d). In addition, we observed that LFA-1, a stabilizing component of the VS, did not become enriched at the interface between the MDDCs and T cells in the absence of virus (data not shown). These data suggest that virus targets cytoplasmic vesicles after entry into MDDCs; however, onward trafficking of virus to the plasma membrane is inhibited by depletion of the target genes, preventing VS formation and reducing efficient *trans*-infection between the DCs and CD4^+^ T cells.

### The integrity of virus-containing vesicles is compromised in *BIN1*- and *RAB7L1*-depleted MDDC cells.

**FIG 4:**
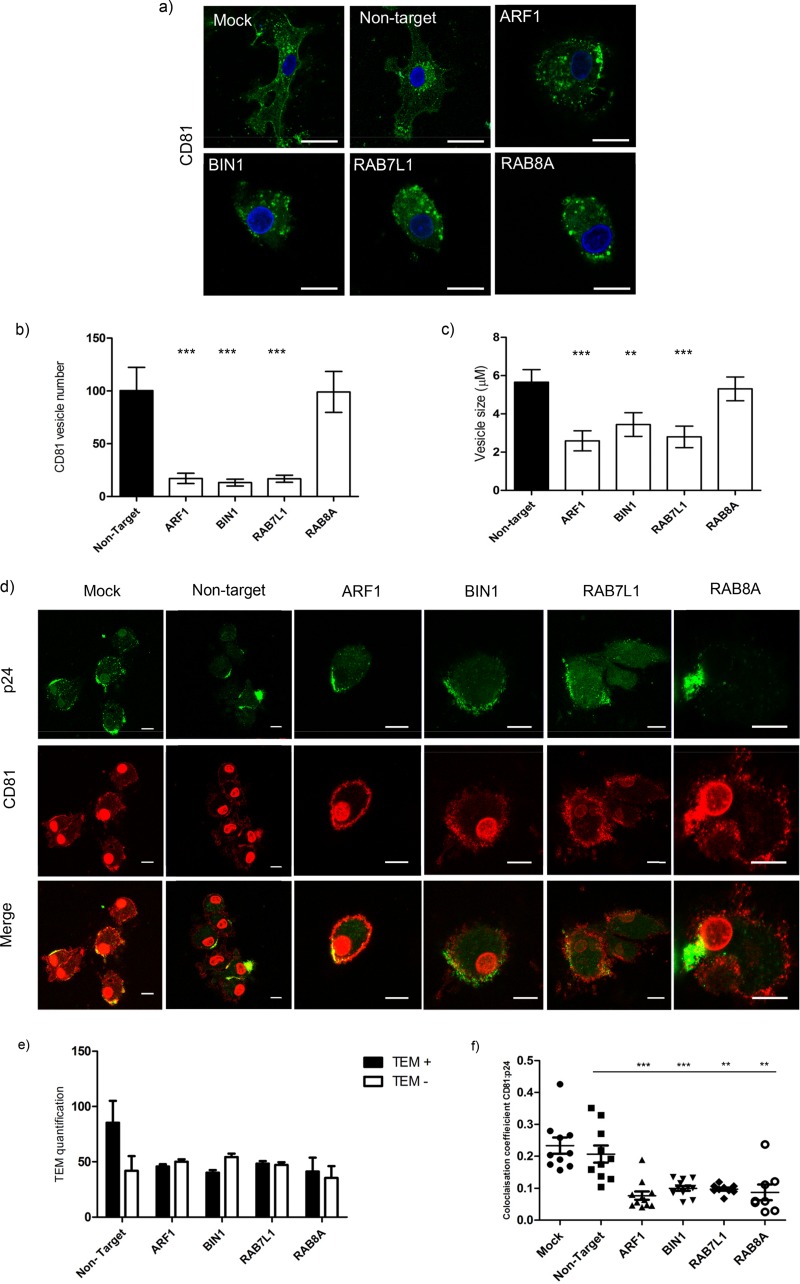
CD81 localization and TEM formation is disrupted in MDDCs transfected with ARF1, BIN1, RAB7L1, and RAB8A siRNA. (a) Effects of target siRNA on CD81 staining and localization in MDDCs. CD81, green; nuclei, blue. Scale, 10 μm. (b) Quantification of CD81 vesicles in target siRNA transfected MDDCs compared to nontarget siRNA controls (*n* = 110 cells, across three independent donors). Means and standard errors of the mean (SEM) are shown. ***, *P* < 0.0005. (c) Average sizes (μm) of CD81-positive vesicles in MDDCs transfected with target siRNA compared to nontarget siRNA (*n* = 150 cells, across three independent donors). Means and SEM are shown. **, *P* < 0.005; ***, *P* < 0.0005. (d) Images of CD81 (red) and HIV-1 p24 Gag (green) in infected MDDCs transfected with nontarget and target siRNA. Images show HIV-1 at 4 h postinfection. Nuclei, red (spherical). Scale, 10 μm. (e) Quantification of CD81 and p24 at tetraspanin-enriched domains (TEMs) in infected MDDCs at 4 h postinfection. The mean percentages of cells with HIV-1 p24 Gag localized at CD81-enriched TEMs are represented by black bars. White bars represent the absence of CD81-enriched TEMs. Mean percentages and SD are shown (*n* = 170 cells, across two independent donors). (f) Colocalization analysis of TEM in siRNA-transfected MDDCs compared to control cells. The colocalization coefficient of CD81 with HIV-1 p24 Gag is shown for each condition. Means ± the SEM are shown (*n* = 11 fields, analyzed over two independent donors). **, *P* < 0.005; ***, *P* < 0.0005.

CD81, a type II transmembrane protein, is one of the main tetraspanins recruited to the host cell membrane during HIV-1 *trans*-infection and is known to colocalize with HIV-1-containing compartments in macrophages and DCs (4, 18, 41). To determine whether target siRNAs altered endogenous CD81 localization in MDDCs, transfected cells were labeled for CD81 (Fig. 4a). In control cells (nontarget siRNA) CD81 is found at the cell periphery with a faint perinuclear staining. In contrast, *ARF1* siRNA underwent a reduction in CD81-positive vesicles that was evident within both the cytoplasm and at the cell periphery. *BIN-1* and *RAB7L1* depletion reduced the CD81 vesicle number and size, whereas no significant difference was observed in cells depleted of *RAB8A* (Fig. 4b and c). In all three cases, an accumulation of CD81 vesicles was observed within the cytoplasm not at the cell periphery (Fig. 4a).

CD81 plays an important role in regulating viral *trans*-infection at the VS, and depletion of the tetraspanin can reduce viral *trans*-infection (42). In light of previous findings, we assessed CD81 localization during HIV-1 infection. As expected, we observed p24 Gag colocalization with CD81 at the cell periphery in CD81 tetraspanin-enriched microdomains (TEMs) at 4 h postinfection in control cells. We observed that the numbers of CD81 p24 Gag TEMs in transfected MDDCs are reduced in *ARF1*-depleted cells. In contrast, in cells transfected with siRNA targeting *BIN1*, *RAB7L1*, and *RAB8A* there were both virus and CD81 at the cell periphery; however, the staining of the TEM was diffuse and lacked the structure of the TEM (Fig. 4d and e). This was confirmed by colocalization data, indicating that CD81 association with p24 was reduced in siRNA-transfected cells (Fig. 4f). Taken together, these data suggest that trafficking of CD81 and p24 Gag to the cell periphery to form the TEM is compromised by knockdown of *ARF1*, *BIN-1*, *RAB7L1*, and *RAB8A*, potentially preventing the efficient *trans*-infection of virus via the VS.

### Retention of virus in endocytic compartments reduces HIV-1 transfer.

We hypothesized that the presence of virus and CD81 in cytoplasmic vesicles and the disrupted trafficking of CD81 and p24 Gag to the plasma membrane by target siRNA was due to retention in endocytic compartments. Therefore, we aimed to trap virus in endosomal derived vesicles to establish whether this directly affects viral *trans*-infection to T cells. MDDCs were treated with endocytic inhibitors prior to infection with R9 virus, and the level of *trans*-infection was measured. We utilized LY294002, a phosphatidylinositol 3-kinase (PI3K) inhibitor known to block macropinocytosis and the formation of early endosomes, and bafilomycin A1, a vacuolar type H^+^-ATPase (V-ATPase) that prevents endosomal acidification.

**FIG 5:**
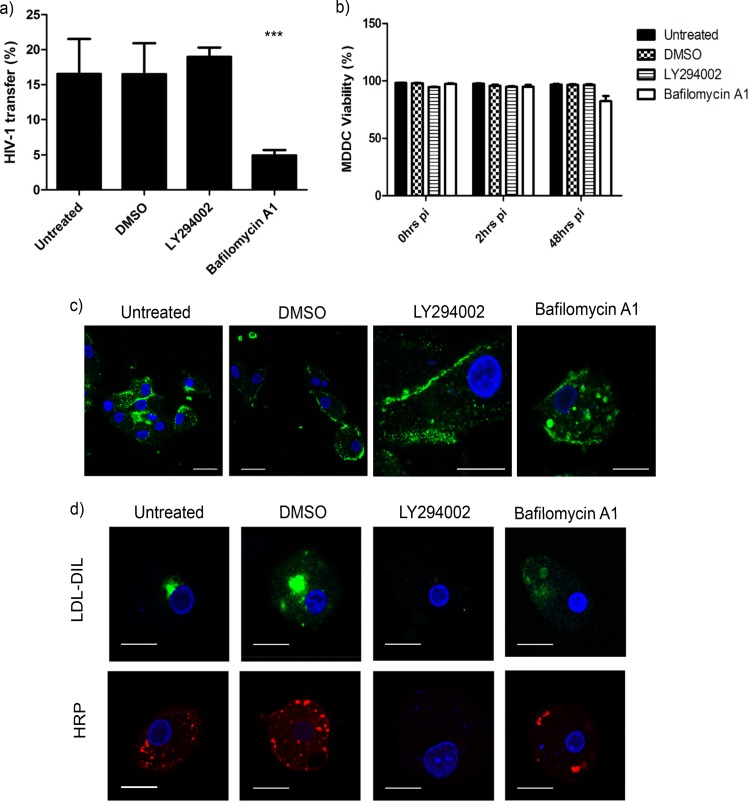
Retention of virus in endocyte-derived compartments reduces HIV *trans*-infection from DCs to T cells. (a) Effect of LY294002 and bafilomycin A1 treatment on HIV-1 transfer. MDDCs were pretreated with inhibitors overnight prior to infection with HIV-1 (R9) before coculture with autologous CD4^+^ T cells for 48 h in triplicate in two independent donors. Mean percentages (%) viral transfer and SD are shown. ***, *P* < 0.0005. (b) Percentage of viable MDDCs after overnight incubation with LY294002 and bafilomycin A1 at 0, 2, and 48 h postinfection (pi). The percentage (%) of reduction in cell viability was assessed by using a Live/Dead stain and flow cytometry analysis. The means and SD are shown. Experiments performed in triplicate in two independent donors. (c) Effect of inhibitor LY294002 and bafilomycin A1 on HIV-1 localization in MDDCs. MDDCs pretreated with inhibitors were infected with HIV-1 for analysis by confocal microscopy. p24 Gag HIV-1 (green) and nuclei (blue) are labeled as indicated. Scale, 10 μm. (d) Effect of LY294002 and bafilomycin A1 on LDL-DIL and HRP uptake into MDDCs. Inhibitors were added overnight before the addition of LDL-DIL (green) and HRP (red). Nuclei are blue. Scale, 10 μm.

Inhibition with LY294002 resulted in a mild increase of HIV-1 *trans*-infection (+20%) compared to dimethyl sulfoxide (DMSO)-treated control cells. However, a 2-fold decrease in *trans*-infection was observed in cells treated with bafilomycin A1, indicating that efficient viral transfer requires a functioning endocytic pathway (Fig. 5a). This reduction was not due to inhibitor toxicity, with MDDCs remaining >80% viable during treatment and subsequent infection (Fig. 5b). To visualize any differences between MDDCs treated with LY294002 and bafilomycin, infected MDDCs were analyzed by confocal microscopy. HIV-1 was concentrated at the cell surface in cells pretreated with LY294002, which is in agreement with previous findings (13). In contrast, virus accumulates inside intracellular vesicles in cells treated with bafilomycin A1 (Fig. 5c), indicating that viral uptake into MDDCs was not inhibited, and that retention within endocytic vesicles reduced *trans*-infection. Controls confirmed that both horseradish peroxidase (HRP) taken into the MDDCs via the fluid phase and the lysosomal marker low-density lipoprotein (LDL) were lost in cells treated with the PI3K inhibitor LY294002, as predicted. In contrast, LDL-DIL labeling was diffuse and cytoplasmic in cells treated with bafilomycin A1, suggesting a block in LDL-DIL uptake by endosomes in the MDDCs. On the contrary, HRP taken up via the fluid phase was less affected, suggesting that unlike LDL, HRP is retained in endocyte-like compartments (Fig. 5d).

HIV-1 did not colocalize with the organelle markers EEA1, Rab5, Rab7, Rab11, LAMP2, or CHMP2B in either siRNA-transfected or bafilomycin A1-treated MDDCs in our experiments, suggesting that these HIV-1-positive compartments may be intermediate vesicles devoid of characteristic markers.

Taken together, these data indicate that HIV-1 transfer is reliant on a functioning endocytic pathway. Blocking virus in endosomal derived compartments results in the accumulation of virus in cytoplasmic vesicles, which in turn reduces viral transfer between MDDCs and T cells, as seen in siRNA-transfected MDDCs. In addition, bafilomycin A1 appears to block LDL-DIL but not HRP or HIV-1 uptake into MDDC, suggesting that HIV-1 is predominantly trafficked to cellular compartments that differ from those utilized by LDL.

### Downstream trafficking from early endosomes is compromised in MDDCs transfected with target siRNA.

Endosomal cargo has one of two fates; it is either recycled back to the cell surface (i.e., transferrin), or it is directed to lysosomes for degradation (i.e., LDL). To confirm that target siRNA is blocking endosomal trafficking in MDDCs, cells were transfected with pooled *ARF1*, *BIN-1*, *RAB7L1*, and *RAB8A* siRNAs and either stained for early endosomes with early endosome antigen 1 (EEA1), incubated with Alexa Fluor-labeled transferrin, or LDL-DIL, to assess the recycling and lysosomal trafficking pathways, respectively.

**FIG 6:**
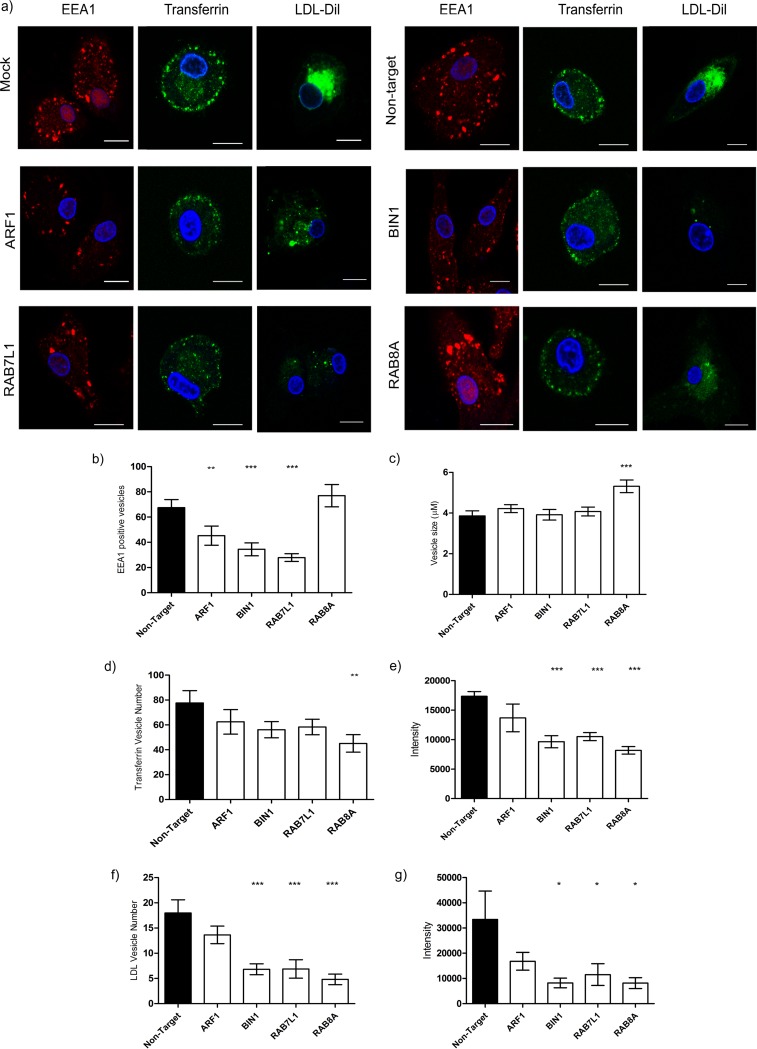
Endocytic trafficking is compromised in BIN1, RAB7L1, and RAB8A transfected MDDCs. The effect of target siRNA on vesicle trafficking in MDDCs was investigated. (a) MDDCs transfected with ARF1, BIN-1, RAB7L1, and RAB8A siRNA for 48 h were labeled with either EEA1 for early endosomes (red, first panel) or incubated with transferrin (green, second panel) for 20 min 37°C or LDL-DIL (green, third panel) for 2 h 37°C. Nontarget siRNA was used a control. Nuclei are indicated in blue. Scale, 10 μm. (b) Quantification of EEA1 vesicles in MDDCs transfected with target siRNA compared to nontarget siRNA control (*n* = 150 cells). Means and SEM from three independent donors are shown. **, *P* < 0.005; ***, *P* < 0.0005. (c) Average EEA1 vesicle sizes (μm) in MDDCs transfected with target siRNA compared to nontarget siRNA control (*n* = 150 cells). Means and SEM from three independent donors are shown. ***, *P* < 0.0005. (d) Quantification of the number of transferrin-positive vesicles under each condition compared to nontarget control (*n* = 150). Means and SEM from three independent donors are shown. **, *P* < 0.005. (e) Measurement of the intensity of transferrin in transfected MDDCs under each condition compared to nontarget control (*n* = 150). Means and SEM from three independent donors are shown. ***, *P* < 0.0005. (f) Quantitative analysis of LDL-DIL containing vesicles (*n* = 120). Means and SEM from three independent donors shown. ***, *P* < 0.0005. (g) Intensity of LDL-DIL in transfected MDDCs compared to nontarget siRNA (*n* = 120). Means and SEM from three independent donors are shown. *, *P* < 0.05.

In nontarget siRNA-transfected MDDCs, EEA1 is seen in numerous vesicles of various sizes throughout the cell. MDDCs transfected with siRNA against *ARF1*, *BIN1*, and *RAB8A* resulted in the formation of abnormal endosomes marked by a decrease in both number and size. *RAB8A* siRNA resulted in more numerous, enlarged vesicles evident at the cell periphery (Fig. 6a to c).

In all instances, labeled transferrin was found localized at the cell periphery, with no discernible differences between control and siRNA-transfected cells (Fig. 6a, second panel). However, a reduction in vesicle number and Alexa Fluor labeling was observed in *BIN1*-, *RAB7L1*-, and *RAB8A*-transfected cells (Fig. 6d and e).

In nontarget siRNA or mock-transfected control cells, LDL-DIL predominantly accumulates in lysosomes in the perinuclear region. In cells transfected with siRNA targeting *ARF1*, LDL has accumulated in various-sized vesicles in the cytoplasm (Fig. 6a, third panel). Knockdown of *BIN-1*, *RAB7L1* resulted in a reduction of LDL containing vesicles within the cells, indicating that the delivery of LDL-DIL to lysosomes is significantly reduced (Fig. 6a and f to g). *RAB8A*-silenced cells were also found to have a reduced number of LDL vesicles; however, diffuse staining is evident within the cytoplasm, suggesting that LDL is taken into the cell but not trafficked within the endolysosomal pathway.

These observations suggest *ARF1* regulates endosomal morphology and vesicle formation and slows LDL trafficking to the perinuclear region but is dispensable for the recycling of transferrin to the plasma membrane. *BIN1* and *RAB7L1* also affect endosomal vesicle formation, resulting in the retention of vesicles at the cell periphery and reducing downstream trafficking from endosomes, as evidenced by a reduction in both transferrin- and LDL-containing vesicles, suggesting that *BIN1* and *RAB7L1* play a role in early endosomal protein trafficking. In contrast, RAB8A depletion appears to increase early endosome size, although trafficking of both LDL and transferrin is also reduced, suggesting that RAB8A action is targeted more downstream, regulating protein trafficking after the cargo has left the endocytic compartment. The disruption of endosomal vesicle trafficking at or after the early endosomal compartment by target siRNA creates a knock-on effect, altering endocytic trafficking to lysosomes and recycling of cargo to the plasma membrane. Taken together, the disruption of TGN-endosomal-plasma membrane trafficking suggests that HIV-1 trafficking from internalized compartments relies on endosomal sorting pathways to traffic to and accumulate at the VS and potentially within VCC at the cell surface.

### HIV-1 *trans*-infection requires retromer complex recycling of cargo.

**FIG 7:**
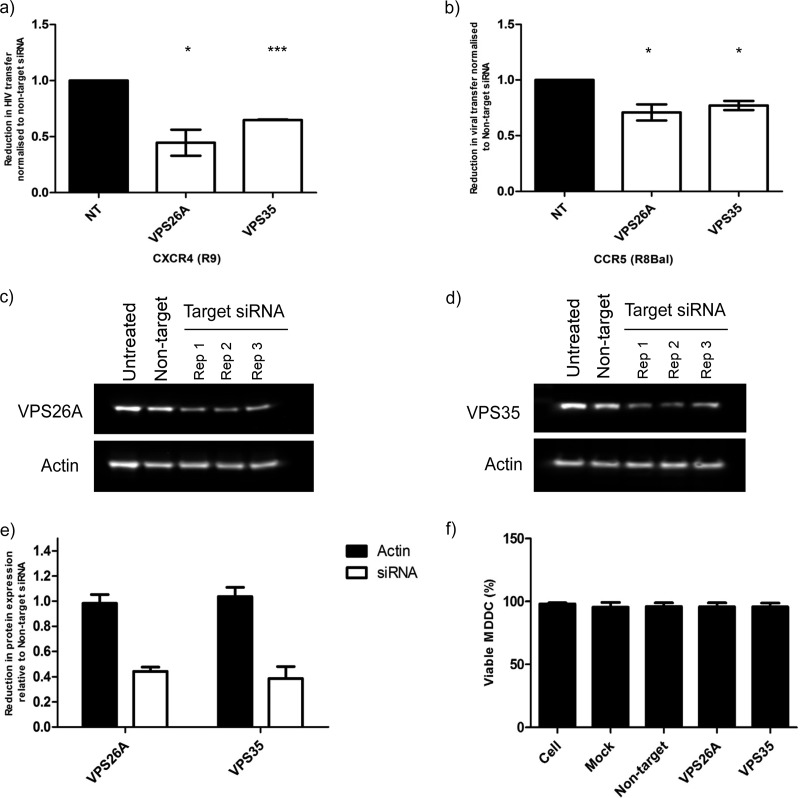
HIV-1 *trans*-infection requires retromer recycling to the plasma membrane. (a and b) The reduction in HIV-1 *trans*-infection between MDDCs and CD4^+^ T cells in MDDCs transfected with VPS26A and VPS35 via siRNA transfection. The reduction in *trans*-infection is normalized to nontarget siRNA for R9 (a) and R8-BAL (b). The means ± the SD are shown (*n* = 4). *, *P* < 0.05; ***, *P* < 0.0001. (c and d) Western blots showing the knockdown of VPS26A and VPS35 in MDDCs, performed in triplicate, compared to untreated cell lysate and nontarget siRNA-transfected MDDCs. Actin used as a loading control. (e) Quantification of protein knockdown of VPS26A and VPS35 in transfected MDDCs relative to the nontarget lane. All lanes are compared to corresponding actin loading control (black bars). The means ± the SD are shown (*n* = 3). (f) Percentage of viable MDDC 48 h after transfection with VPS siRNA compared to controls. The means and the SD are shown (*n* = 2).

Based on the findings that trafficking between key endosomal compartments is compromised in siRNA-targeted cells, which in turn reduces HIV-1 trafficking to the VS, we wanted to confirm whether *trans*-infection of HIV-1 was reduced when trafficking to the plasma membrane from the TGN or early endosomes is compromised. Several of our selected genes play a key role in endosomal sorting to the TGN and plasma membrane, with *RAB7L1* specifically involved in retromer activity. In addition, a proportion of transferrin and its receptor are recycled in a retromer-dependent manner to the plasma membrane (43). The retromer has also been found to play a key role in HIV-1 Env trafficking and viral assembly (44). Thus, we decided to investigate the role of the retromer complex in *trans*-infection using siRNA targeting key components, VPS26A and VPS35, of the retromer complex. HIV-1 *trans*-infection was significantly reduced in MDDCs transfected with each of the retromer siRNAs (Fig. 7a and b) from 25 to 50%. A more marked reduction was evident in cells infected with CXCR4-tropic strain of the virus. A protein knockdown of approximately 60% was confirmed for both VPS26A and VPS35 (Fig. 7c to e), and no reduction in cell viability was evident resulting from siRNA transfection of the VPS genes (Fig. 7f). Therefore, we were able to confirm endosomal sorting between the TGN and the plasma membrane is required for HIV-1 *trans*-infection.

## DISCUSSION

DCs perform an essential role in the transmission of HIV-1 to target CD4^+^ T cells, promoting the spread of infection. Although there have been numerous investigations into the role of DCs in *trans*-infection, the cellular trafficking pathways exploited by HIV-1 remain unclear. The identification of host cell factors and intracellular pathways exploited by HIV-1 to aid *trans*-infection of T cells will facilitate the development of novel therapies and may reduce initial transmission of HIV-1.

In this study, we identified a number of host factors involved in *trans*-infection of HIV-1 from DCs to T cells. By conducting an siRNA screen targeting membrane trafficking proteins, we identified four genes involved in efficient *trans*-infection from DCs to T cells. Although one similar shRNA/siRNA screen has been conducted investigating the role of membrane trafficking in HIV-1 *trans*-infection, none to date has focused on the genes we identified in the present study. In a recent shRNA screen, Menager and Littman (13) also identified *ARF1*, *ARF6*, and *ARPC1B* as reducing viral transfer and *CLTC*, *CLTB*, and *AP2M1* as enhancing viral transfer; however, the ability to draw direct comparisons between the two studies is complicated by the fact that these authors used shRNA technology in a screen that targets a different gene library, several of them not included in the siRNA screen we utilized. The study then proceeds to concentrate on TSPAN7 and DNM2 and their role in *trans*-infection at the cell surface, whereas we focused on the trafficking of internalized virus. In a study using an identical siRNA screen, Wen et al. identify a number of common genes such as *RAB7L1*, *AP1M1*, *BIN-1*, *ARPC1B*, *DIAPH1*, *ARF6*, *WASF1*, *CLTC*, and *VAV2* required for HIV-1 and M-PMV virus release from HeLa cells (45). Overall, there is a high consistency of hits between previous screens conducted in DCs and our own membrane trafficking screen, verifying our findings.

Our initial siRNA screen shows that knocking down genes associated with clathrin-coated vesicle formation enhanced *trans*-infection; this suggests that restricting viral uptake into MDDCs and retention of virus on the cell surface promotes HIV-1 *trans*-infection. It has been previously demonstrated that soluble CD4 protein is able to inhibit infection, suggesting that virus particles bound to the surface of the MDDCs were the main source of *trans*-infection (12). In support of this model, Menager et al. demonstrated that DNM2 and TSPAN7, which coordinate actin nucleation and stabilization, had roles in restricting endocytosis and maintaining virus on cellular dendrites enabling transfer (13, 46). On the other hand, there is compelling evidence for the model that HIV-1 is sequestered in plasma membrane-derived invaginated compartments induced upon HIV-1 uptake (33). From this compartment, viral particles can be released to the VS to initiate *trans*-infection (15–17). We initially identified nine genes from the siRNA screen that reduced *trans*-infection. These genes were predominantly associated with cytoplasmic, membrane-bound vesicles with direct involvement in vesicle-mediated transport and membrane organization, thus supporting a requirement for membrane-bound vesicles in HIV-1 *trans*-infection. These results provide evidence for both viral transmission via the cell surface and trafficking via intracellular compartments to promote *trans*-infection in MDDCs.

Although the use of primary MDDCs and CD4^+^ T cells is a representative model of HIV-1 *trans*-infection, employing methods such as siRNA transfection within established MDDCs has its limitations. Generally, 50% transfection efficiency is achieved, which in turn does not completely block reduction of *trans*-infection within these cells. However, partial knockdown is still capable of producing a strong phenotype, and the study of these pathways in primary cells is essential to uncovering underlying mechanisms of *trans*-infection and is critical for investigating and identifying such cellular processes.

Here, we concentrate on studying genes required for efficient viral *trans*-infection and therefore aim to investigate how internalized virus is trafficked to the VS. We demonstrate that the reduction in viral *trans*-infection observed from the depletion of four genes—*ARF1*, *BIN1*, *RAB7L1*, and *RAB8A*—is due to the apparent retention of virus in intracellular vesicles and a reduction in virus accumulation at the VS between DC and T cells. MDDCs can capture and store HIV-1 virions in invaginations at the plasma membrane (9, 15). Live imaging shows that viral puncta are trafficked into enclosed intracellular compartments (47); whether these compartments are enclosed or remain accessible to the cell surface is still a matter of debate (15, 16). The integrity and formation of intracellular compartments are believed to be regulated by membrane trafficking processes (17). Based on these data, we propose that the reduction in VS formation observed in siRNA-treated MDDCs disrupts the regulation and trafficking of intracellular compartments, resulting in the retention of viral particles within intracellular vesicles, preventing onward trafficking to the VS and therefore inhibiting viral *trans*-infection.

VS formation and HIV-1 spread relies on the interaction of MDDCs and recipient T cells, triggering the active polarization of organelles and cell surface proteins. One such component, LFA-1, has been shown to induce T-cell polarization toward the VS to induce efficient viral T-cell-to-T-cell spread (48). In the context of VS formation between DCs and T cells, it has been reported that cell-to-cell contacts are not increased by the presence of HIV-1 and that the formation of the VS was decreased by 60% when the interaction between ICAM-1 and LFA-1 was blocked (49). Our findings agree with these data. We also observed several T cells interacting with HIV-1-infected siRNA-transfected MDDCs; however, accumulation of LFA-1 at the VS was only evident in the presence of HIV-1 p24 Gag. These data suggest that by blocking trafficking of HIV-1 to the cell periphery, enrichment of LFA-1 at the MDDC-T-cell interface is also prevented, restricting VS formation. It may be the case that virus alone is not the only trigger for VS formation, and it is plausible that by blocking trafficking of HIV-1 to the cell surface in MDDCs we may also be preventing the recruitment of other key components to form efficient VS.

We also observed the retention of endogenous CD81 in cytoplasmic vesicles and a reduction in localization at the cell periphery. In addition, at 4 h postinfection, TEMs are reduced or disrupted, potentially affecting the recruitment and budding of HIV-1 at the VS. The tetraspanin CD81 colocalizes with HIV-1 within VCC (4, 18, 41) and accumulates at the VS, promoting viral *trans*-infection, preventing cell-to-cell fusion, and providing a platform for viral budding (50, 51). Our results are consistent with these findings, suggesting that trafficking of CD81 within MDDCs to the plasma membrane and recruitment to TEMs, along with HIV-1, are required for *trans*-infection. This is supported further by a study showing that blocking CD81 with specific antibodies reduces VS formation (52). Conversely, Krementsov et al. showed that direct depletion of CD81 actually enhances viral transmission between HeLa and Jurkat cells (53). The different outcomes observed in these studies may reflect the different methods and cell types employed to target CD81 and reduce its presence at the VS. Our data support the former approach, where CD81 is still present within the cell but is prevented from forming functioning TEMs at the cell periphery, whereas actual depletion of CD81 from cells may have a number of downstream effects, altering normal cell function.

Overall, we demonstrate that targeting host factors that regulate endocytic compartments and vesicle trafficking to the plasma membrane within MDDC results in the disruption of trafficking of CD81 and virus to the VS reducing *trans*-infection.

Our results show that upon disruption of target genes, protein trafficking to lysosomes and recycling of transferrin to the plasma membrane is reduced: this suggests that endosomal sorting and recycling to the plasma membrane are closely linked to *trans*-infection in MDDCs.

In conjunction with other ARF proteins, ARF1 plays an important role in the regulation of recycling endosome morphology and recycling pathways; however, depletion of the gene was not found to directly affect the recycling of transferrin receptor (54, 55). Depletion of ARF1 in our study is consistent with this role in protein recycling in MDDCs, altering endosomal morphology but not affecting recycling of transferrin to the plasma membrane. In the context of infection, HIV-1 ability to mediate the downregulation of MHC-1 is achieved by targeting AP-1 and ARF1 activity (56), resulting in the accumulation of MHC-1 in the TGN or endosomes (57). HIV-1 Vpu also targets the same pathway (58, 59) to counteract tetherin, which is known to block the release of progeny virus from the cell (60). These data, in conjunction with the fact that ARF1 binding partner AP1M1 was originally identified as a potential gene required for *trans*-infection in our screen, support the idea that the same recycling pathway could be utilized for the successful *trans*-infection of HIV-1 in DCs. Depletion of ARF1 is likely to impact the morphology of virus-containing compartments and the recycling of internalized virus to the cell surface, which in turn reduces the accumulation of virus at the VS and therefore *trans*-infection.

BIN-1 is a key player in membrane remodeling during endocytosis and endosomal sorting and is essential for the formation of plasma invaginations in muscle tissue (61). BIN-1 mutants were found to both impair membrane tabulation and cause compact membrane curvature (62). Our findings support these data: depletion of BIN-1 in MDDCs reduces endosomal size, producing small round vesicles and preventing downstream trafficking. A role for BIN-1 in HIV-1 infection is supported further by a study that identifies the upregulation of BIN-1 in CD4^+^ and CD8^+^ T cells from *ex vivo* patients (63). Based on this, we propose that BIN-1 is required for the efficient formation and function of plasma membrane invaginations and endosomal sorting that assist the trafficking of HIV-1 to the VS.

RAB7L1 also plays a role in intracellular trafficking and the endosomal sorting of lysosome-bound membrane proteins (64). Again, our results support a similar role for RAB7L1 in MDDCs; the transport of both LDL and transferrin was impaired in RAB7L1-depleted MDDCs, suggesting that trafficking from endosomal compartments is compromised. The finding that RAB7L1, along with AP1M1, is involved in HIV-1 Gag trafficking and virion budding in the activated macrophage cell line MM6 and in CD4^+^ Jurkat cells (65) supports a role for RAB7L1 in the recruitment of HIV-1 particles in MDDCs to the VS to assist viral budding at the cell surface.

RAB8A is known to control vesicular transport and promote membrane protrusions, which can be inhibited by blocking membrane recycling (66), in agreement with our findings. Knockdown of RAB8A by siRNA in previous studies was found to inhibit HIV-1 replication in HeLa P4/R5 cells and directly interact with *nef*, *env*, and *gag-pol* (67). Therefore, it seems plausible that the depletion of RAB8A in MDDCs inhibits membrane recycling and therefore membrane protrusions, reducing HIV-1 *trans*-infection. The data also support the idea that HIV-1 taken up by MDDCs could rely on the same recycling pathways to traffic to the cell membrane to accumulate virus at the VS.

The data presented here clearly point to a role for endocytic recycling pathways in HIV-1 *trans*-infection; therefore, we investigated the retromer complex implication in the trafficking of HIV-1 to the VS. Retromer-dependent protein sorting pathways provide an opportune target for a variety of viral and bacterial pathogens (68, 69). For instance, HIV-1 envelope protein and herpesvirus saimiri, a T-lymphotrophic tumor virus, bind the retromer to aid infection and viral release (44, 70), whereas influenza A M2 protein escapes degradation via transportation from early endosomes to the TGN (71). Our data confirm a role for the retromer in DC-mediated HIV-1 *trans*-infection and exploitation of recycling pathways by the virus to achieve efficient transfer between cells.

**FIG 8:**
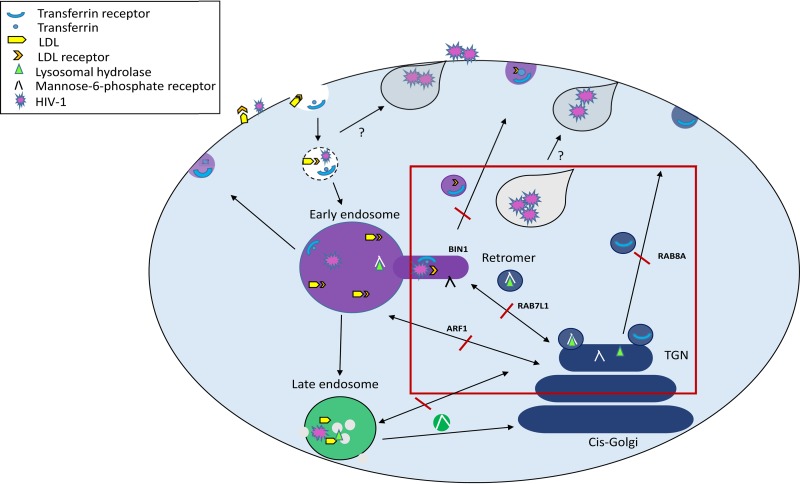
Model for the roles of ARF1, BIN1, RAB7L1, and RAB8A in the endocytic pathway and vesicle formation in MDDCs. Molecules are internalized from the cell surface via endocytic vesicles that fuse with each other or existing endocytic vesicles to form early endosomes. The budding of vesicles containing cargo from early endosomes to the plasma membrane and *trans*-Golgi network (TGN) requires the activity of BIN1. TGN vesicles bud from the TGN surface and either fuse with each other or endocytic compartments. The TGN is responsible for sorting receptors from degradative compartments and delivers newly synthesized lysosomal enzymes in the form of lysosomal hydrolase via the mannose-6-phosphate receptor. Both transferrin and LDL are taken into the cell via clathrin-receptor-mediated endocytosis. Transferrin and its receptor are recycled from early endosomes back to the plasma membrane. LDL is trafficked directly to lysosomes prior to release into the cytoplasm. The dynamic retrograde transport of vesicles between the TGN and endocytic compartment and the plasma membrane via the retromer and other trafficking pathways depends on the activity of ARF1, RAB7L1, and RAB8A. HIV-1 *trans*-infection between MDDCs and CD4^+^ T cells requires a homeostatic balance of the endocytic pathway. By blocking trafficking of molecules between early endosomes and the TGN and onward polarized transport of cargo to the plasma membrane, HIV-1 *trans*-infection is inhibited. Depletion of targeted proteins results in the accumulation of HIV-1 in intracellular vesicles that are unable to traffic to the virological synapse.

We hypothesize that VCC and VS formation is dependent on the retromer-dependent endocytic-TGN-plasma recycling pathway. By exploiting the retromer pathway, internalized viral particles can be subverted to the plasma membrane, where virus becomes sequestered to promote VS formation and enable *trans*-infection between MDDCs and T cells (Fig. 8).

DCs are among the most important cellular targets in early HIV-1 transmission. HIV-1 is thought to accumulate in “viral endosomes” where the virus is able to exploit a pathway essential for the delivery of components to the immunological synapse and activation of T cells (4). Uptake into DCs using this method not only allows efficient *trans*-infection to target CD4^+^ T cells but also evades detection by the immune system (27), the importance of which was shown *in vivo* using a humanized mouse model (10, 11).

By using high-throughput siRNA screening, we were able to identify *ARF1*, *BIN1*, *RAB7L1*, and *RAB8A* as essential for endosomal trafficking between the TGN and early endosomes and coordinated transport to the plasma membrane in a retromer-dependent manner. Thus, we identify key cellular trafficking proteins exploited by HIV-1 in DCs to efficiently disseminate virus to target T cells promoting *trans*-infection. A better understanding of the role of these proteins in viral transfer to T cells may serve as potential candidates for targeted therapy to control the transfer of HIV-1 between DCs and T cells *in vivo*.

## MATERIALS AND METHODS

### Ethics statement.

Peripheral blood mononuclear cells (PBMCs) were derived from buffy coats obtained from healthy blood donors, anonymously provided by the Welsh Blood Service, UK. Written informed consent for the use of buffy coats for research purposes was obtained from blood donors, and the use of patient samples and procedures were approved by the local research ethics committee at Cardiff University.

### Cells.

Primary cells were isolated from PBMCs of healthy blood donors using magnetic bead selection (Miltenyi Biotech). CD14^+^ monocytes were differentiated into immature monocyte-derived dendritic cells (MDDCs) with IL-4 and granulocyte-macrophage colony-stimulating factor, as described previously (72, 73).

CD4^+^ T cells were isolated using CD4^+^ magnetic beads (Miltenyi Biotech), maintained in the presence of IL-2, and activated 4 days before use with 2 μg/ml PHA. SUP-T1 T lymphoblasts and 293T human embryonic kidney (HEK) cells (obtained from NIH AIDS Research and Reference Reagent Program) were maintained in supplemented RPMI 1640 or Dulbecco modified Eagle medium, respectively.

### Viral stock production.

Viral stocks were produced by transfection of HEK293T cells with calcium phosphate DNA precipitation of proviral plasmids encoding full-length HIV-1×4 and R5 provirus, pR9 and pR8BAL, respectively (the plasmids were provided by D. Trono D [EPFL, Lausanne, Switzerland]). Infectious titers were determined by titration onto SUP-T1 cells and quantification of HIV-1 p24 Gag by enzyme-linked immunosorbent assay using a Lenti-X p24 rapid titer kit (Clontech).

### Antibodies and reagents.

HIV-1 p24 was detected using anti-HIV-1 core antigen antibody-FITC (KC57-FITC; Beckman Coulter), and actin labeled with Cytopainter Phallodin-iFluor-555 (Abcam). Protein knockdown was detected by immunoblotting using rabbit anti-ARF1, anti-BIN1, anti-RAB8A, mouse anti-Rab7L1 (Abcam), and actin (Merck) antibodies, followed by secondary HRP-conjugated goat anti-rabbit and anti-mouse antibodies (Dako). Confocal microscopy was carried out using the primary antibodies anti-human CD81-APC (BD), anti-EEA1, anti-CHMP2B, anti-LAMP1, anti-Rab7, anti-Rab11, anti-Rab5 (Abcam). HRP uptake was detected using anti-HRP (Jackson Immunolaboratory). All unlabeled primary antibodies were detected with secondary anti-rabbit Alexa Fluor 546 (Life Technologies). The pharmacological inhibitors LY294002, bafilomycin A1, and indinivir (Sigma-Aldrich) were used at 50 μM, 0.5 μM, and 2 μg/ml, respectively.

### RNAi screen in MDDCs.

An siRNA screen was performed using a commercially available SMARTpool ON-TARGET library containing 140 membrane trafficking genes (Dharmacon-GE Healthcare). MDDCs (1 × 10^5^ cells/well) seeded in 96-well plates were reverse transfected twice, 24 h apart, with 200 nM pooled siRNA or with control siRNA (SMARTpool ON-TARGET nontarget siRNA; Dharmacon-GE Healthcare) using HiPerFect transfection reagent (Qiagen) in serum-free media. After 48 h, the MDDCs were infected with 20 to 30 ng of p24 Gag HIV-1 R9 by spinoculation and cocultured with SUP-T1 or CD4^+^ T cells prestained with Celltrace Far Red (Invitrogen) in the presence of indinivir at 2 μg/ml (Sigma) for a further 48 h, as previously described (72).

### Flow cytometry.

Phenotyping of primary cells was performed by washing MDDCs and CD4^+^ T cells in ice-cold buffer (phosphate-buffered saline [PBS], 0.5% bovine serum albumin [BSA]) before staining for HLR-DR, CD209, CD83, or CD14-APC (BD). SUP-T1 and autologous T cells were labeled with CD4 and CD3-APC (BD). Cell viability was assessed using Live/Dead stain at 1:1,000 (Life Technologies) in PBS according to the manufacturer’s instructions. Infected MDDCs and CD4^+^ T cells were fixed in 2% paraformaldehyde (PFA) and stained for HIV-1 p24 Gag-FITC after permeabilization with 1× PhosFlow buffer (BD). Stained samples were washed twice before measurements were taken using a FACSCalibur (Becton Dickinson) Canto II and analyzed with FlowJo V10 software (FlowJo, LLC).

### Transfer assay.

MDDCs (1 × 10^5^ cells/well) were reverse transfected twice, 24 h apart, with 200 nM pooled or individual siRNA using HiPerFect transfection reagent (Qiagen) in serum-free media. After 48 h, the MDDCs were infected with 5 to 10 ng of p24 Gag HIV-1 R9 or 2 to 5 ng of p24 Gag HIV-1 R8BAL by spinoculation for 2 h and then cocultured with CD4^+^ T cells prestained with Celltrace Far Red (Invitrogen) at 37°C for a further 48 h.

### Western blot analysis.

At 72 h posttransfection, the cells were lysed with 1× cell lysis buffer (Cell Signaling), and supernatants were harvested and reduced. Cell lysates were separated on a 4 to 12% SDS-PAGE gel and run next to a PAGEruler (Thermo Fisher) and then analyzed by Western blotting, followed by enhanced chemiluminescence detection and densitometry analysis (MyImage analysis; Thermo Scientific).

### Uptake assays.

Transfected MDDCs were incubated with HRP (Sigma) at 10 mg/ml for 1 h at 4°C, fixed on coverslips using 2% PFA, and labeled using the indicated antibodies.

Transfected MDDCs (1 × 10^5^) were seeded onto poly-l-lysine-coated coverslips and placed at 4°C for 10 min prior to the addition of either 12 μg/ml LDL-DIL (Life Technologies) for 4 h or 25 μg/ml transferrin Alexa Fluor 488 (Life Technologies) for 30 min, both at 37°C. Cells were fixed in 1% PFA and nuclei labeled with TOPRO-3 (Life Technologies).

### Inhibition assays.

Inhibitors LY294002 (50 μM) and bafilomycin A1 (0.5 μM) were added to MDDCs prior to and during infection with R9 HIV-1. DMSO was used as a control at equal concentrations. Cells were either seeded on coverslips and fixed in 2% PFA for confocal imaging, or they were washed and cocultured with CD4^+^ T cells for 48 h at 37°C for analysis via flow cytometry.

### Virological synapse assay.

MDDCs transfected with siRNA were infected with HIV-1 for 2 h prior to incubation with CD4^+^ T cells on poly-l-lysine-coated coverslips at 1:1 ratio for 40 min at 37°C. Fixed cells (2% PFA) were labeled for actin and p24 Gag-FITC and viewed on the confocal microscope. Virological synapse formation was counted if an accumulation (∼50% or greater) of p24 Gag was evident at or adjacent to the junction between T cells and MDDCs. T cells were identified by their smaller size and less cytoplasmic content in comparison to larger MDDCs.

### Confocal immunofluorescence.

Cells were adhered to poly-l-lysine coverslips (Corning), fixed in 2% PFA, permeabilized with 0.05% saponin, and stained with indicated primary antibodies in PBS–0.2% BSA–0.05% saponin, followed by Alexa Fluor-labeled secondary antibodies (1:400) when necessary. TOPRO3 in PBS (1:1,000) was used to stain nuclei (Life Technologies). Confocal microscopy analysis was carried out using a Zeiss LSM710 with a 100× oil objective with 488, 546, and 633 nm acquired sequentially using ZENlite software (Zeiss). All confocal images represent a single plane. Colocalization analysis was performed using Zenlite software (Zen Blue) with the colocalization function.

### Bioinformatics-protein interrelationship mapping.

RNAi screen candidates were enriched using DAVID to identify significant gene ontology (GO) terms, and a protein-protein interaction network was visualized using the EnrichmentMap (Bader Lab) plug-in for Cytoscape 3.3.3. The top five significant values are reported. The minimum confidence score was set at 0.005 (74–77).

### Image analysis.

Image analysis was performed using ImageJ software (National Institutes of Health) and analyzed with Excel software (Microsoft). A macro was designed to apply a set scale to all images, followed by the color threshold, to eliminate any background staining, and the particle analysis function was applied to quantify vesicles. Pixels were converted to μm using the set scale.

### Statistics.

Data were analyzed using a two-sample *t* test, comparing nontarget to targeted siRNA samples. A one sample *t* test was used to compare siRNA transfer assays across donors. *P* values of <0.05, <0.005, and <0.0005 (indicated by asterisks [*, **, and ***, respectively] in the figures) were considered significant. Data were analyzed using Prism (GraphPad) software.
